# Recombinogenic Conditions Influence Partner Choice in Spontaneous Mitotic Recombination

**DOI:** 10.1371/journal.pgen.1003931

**Published:** 2013-11-07

**Authors:** James D. Cauwood, Anthony L. Johnson, Alexander Widger, Rita S. Cha

**Affiliations:** Stem Cell Biology and Developmental Genetics, National Institute for Medical Research, MRC, The Ridgeway, London, United Kingdom; The University of North Carolina at Chapel Hill, United States of America

## Abstract

Mammalian common fragile sites are loci of frequent chromosome breakage and putative recombination hotspots. Here, we utilized Replication Slow Zones (RSZs), a budding yeast homolog of the mammalian common fragile sites, to examine recombination activities at these loci. We found that rates of *URA3* inactivation of a *hisG-URA3-hisG* reporter at RSZ and non-RSZ loci were comparable under all conditions tested, including those that specifically promote chromosome breakage at RSZs (hydroxyurea [HU], *mec1Δ sml1Δ*, and high temperature), and those that suppress it (*sml1Δ* and *rrm3Δ*). These observations indicate that RSZs are not recombination hotspots and that chromosome fragility and recombination activity can be uncoupled. Results confirmed recombinogenic effects of HU, *mec1Δ sml1Δ*, and *rrm3Δ* and identified temperature as a regulator of mitotic recombination. We also found that these conditions altered the nature of recombination outcomes, leading to a significant increase in the frequency of *URA3* inactivation via loss of heterozygosity (LOH), the type of genetic alteration involved in cancer development. Further analyses revealed that the increase was likely due to down regulation of intrachromatid and intersister (IC/IS) bias in mitotic recombination, and that RSZs exhibited greater sensitivity to HU dependent loss of IC/IS bias than non RSZ loci. These observations suggest that recombinogenic conditions contribute to genome rearrangements not only by increasing the overall recombination activity, but also by altering the nature of recombination outcomes by their effects on recombination partner choice. Similarly, fragile sites may contribute to cancer more frequently than non-fragile loci due their enhanced sensitivity to certain conditions that down-regulate the IC/IS bias rather than intrinsically higher rates of recombination.

## Author Summary

Chromosome rearrangements are frequently associated with human cancers. Such rearrangement can result from a DNA break followed by an erroneous repair. Mammalian common fragile sites are one of the most extensively studied naturally occurring breakage prone regions of the genome. It has been proposed that fragile sites are recombination hotspots and that increased recombination activity at these loci contribute to cancer. We examined this hypothesis using a model organism, budding yeast *Saccharomyces cerevisiae*, where a homolog of the mammalian common fragile sites has been identified. Unexpectedly, our results showed that the rate of recombination at the fragile sites was not any higher than non fragile sites, even under the conditions that promoted chromosome breakage at the fragile sites. However, we found that the frequency of loss of heterozygosity (LOH) and translocation, the type of recombination outcomes known to contribute to cancer, to be significantly elevated at fragile sites under certain conditions. These findings suggest that the fragile sites might indeed contribute to cancer more frequently than non-fragile loci, but the reason for this is likely to be due the nature of the recombination outcome(s) rather than higher rates of recombination.

## Introduction

Accidental DNA double strand breaks (DSBs) arise during unperturbed proliferation. Such “endogenous” or “spontaneous” chromosome breakage does not occur randomly throughout the genome, but at specific loci, often referred to as fragile sites. Fragile sites have been observed in organisms ranging from bacteria to mammals, suggesting that they might be a ubiquitous feature of the genome [1], [2], [3], [4]. Evidence points to the existence of multiple types of fragile sites that are distinguishable from one another based on its structure, function, and/or genetic requirement(s) for its stability [5], [6], [7].

Mammalian fragile sites are one of the most extensively studied naturally occurring breakage prone regions of the genome. They are classified as either “rare” or “common”, depending on their incidence among general population [4]. Rare fragile sites are found in less than 5% of the population. In most cases, rare fragile sites are tri-nucleotide repeats, expansion of which has been linked to conditions such as Fragile X-syndrome and Huntington disease [8]. In contrast, common fragile sites are present in all individuals and can be found on every chromosome, indicating that they are a normal component of the chromosome. Common fragile sites extend over large regions of the genome, from several hundred kilobases (kb) to over 1 megabase (Mb) with breaks or gaps occurring throughout these regions. There is no sequence determinant that defines common fragile sites [4], [9].

Studies have implicated a link between common fragile sites, genome instability, and cancer [9]. For instance, some fragile sites have been shown to be loci of frequent chromosome deletions, translocations, and/or viral genome integration [10], [11] as well as oncogenic chromosomal rearrangements (e.g. [12]). Combining these with the observations that some fragile sites in model organisms exhibit elevated rates of recombination, it has been proposed that mammalian common fragile sites are recombination hotspots and that increased recombination activities at these loci contribute to cancer.

Replication Slow Zone (RSZ) is a type of fragile site in budding yeast and a putative homolog of the mammalian common fragile sites. It was identified as loci of preferred chromosome breakage following inactivation of Mec1, the budding yeast homolog of ATR, where high levels of replication dependent single stranded DNA (ssDNA), a precursor to DSBs, accumulate [3], [13]. Like its mammalian counterpart, RSZs are relatively large (∼10 kb), and appear to be a normal component of the chromosome. Other similarities between the two include; (i) timing of their replication during normal S-phase, which occurs late, (ii) their sensitivity to mild replication stress and inactivation of the ATR/ATM family kinases, and (iii) the lack of a defining sequence determinant(s) [3], [14], [15], [16]. The mammalian ATR/ATM and their budding yeast homologs Mec1/Tel1 are conserved signal transduction proteins best known for their roles in S-phase and DNA damage checkpoint responses [17]. In addition, they also play essential roles in a number of fundamental DNA and chromosomal processes including genome duplication, meiotic recombination, and DNA repair (e.g. [3], [18], [19]).

Here, we utilized RSZ as a model to test the proposal that mammalian common fragile sites are recombination hotspots. Unexpectedly, we found that recombination rates at RSZ and non RSZ loci were comparable under all conditions tested, indicating that RSZs are not recombination hotspots. Based on these and other observations, we propose a model whereby regulation of the nature of the recombination outcome(s), irrespective of the overall recombination activity, may play a key role in controlling genome rearrangements.

## Results

### Experimental system

#### RSZ

During normal S phase, replication forks slow down as they enter the ∼10 kb regions located between active replication origins, referred to as Replication Slow Zones (RSZs). The mechanism and/or significance of the fork slowing down remains unknown, but is mechanistically linked to fragility at these loci [3]. In addition to prolonged fork stalling, chromosome breakage at RSZs requires the onset of Topoisomerase II and condensin mediated mitotic chromosome condensation/compaction [20]. Inactivation of Mec1, high temperature, and/or exposure to a modest concentration of hydroxyurea (HU) promotes RSZ breakage [21]. HU is an inhibitor of the ribonucleotide reductase (RNR) complex and causes a dose dependent reduction in intracellular dNTP levels. Consistent with the notion that RSZ breakage is promoted by insufficient dNTP pools, the breakage is suppressed by *sml1Δ*, a mutation shown to increase intracellular dNTP levels [22]. Evidence indicates that *sml1Δ* suppresses RSZ breakage by bypassing the fork slowing that normally occurs at these loci [3].

#### Recombination tester construct

**Figure 1:**
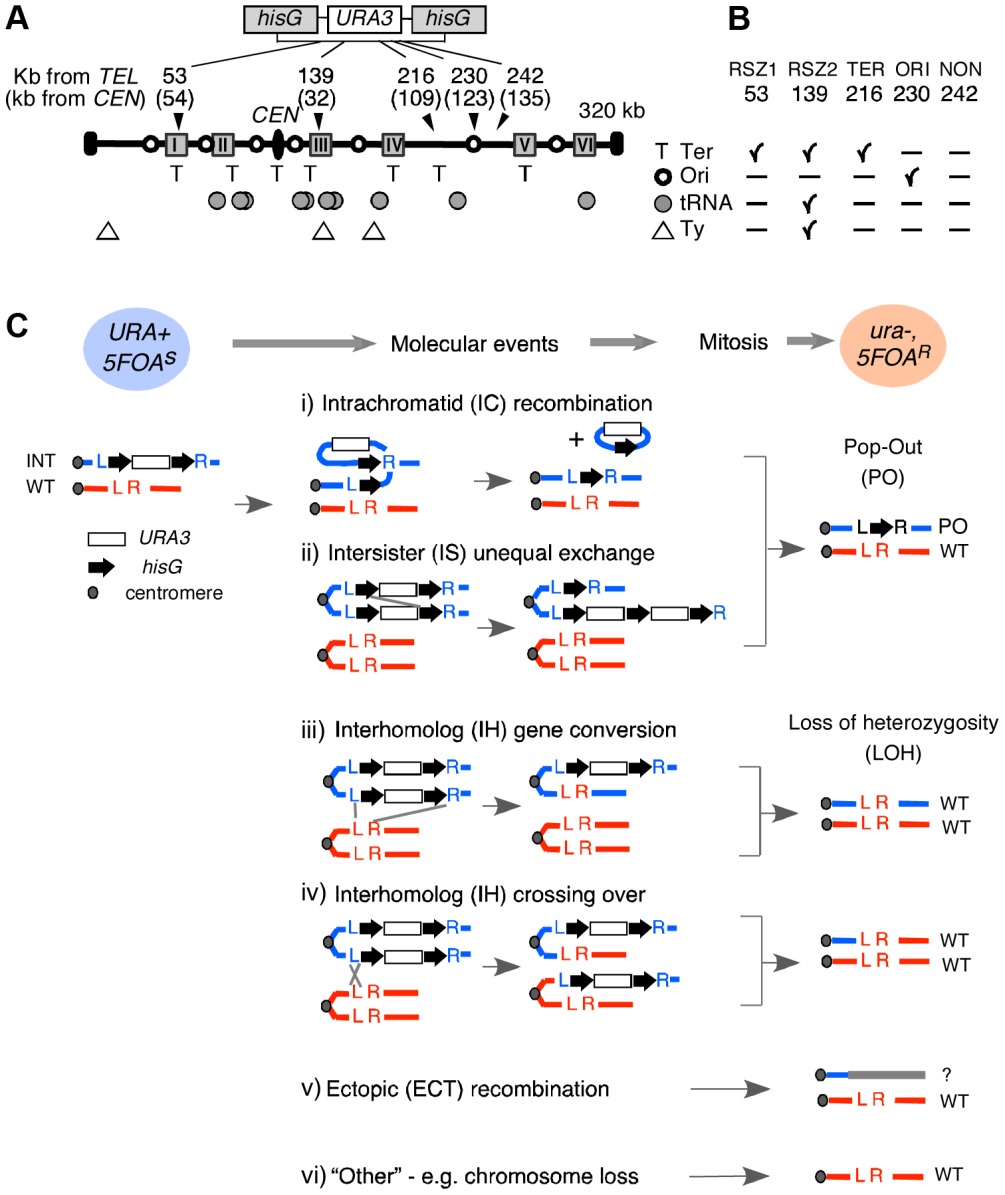
Experimental system. **A.** Recombination activity at five different loci in chromosome III (ChrIII) was assessed by monitoring *URA3* inactivation in *hisG-URA3-hisG* reporter introduced at each of the five indicated site. The “kb from TEL” and “kb from CEN” are distances from the left telomere and the centromere (CEN) in kb, respectively. Grey boxes represent the six RSZs referred to as I through IV in ChrIII [3]. T: replication fork termination site; Open circle: active replication origin; Grey circle: tRNA gene; Triangle: Ty element. **B.** Summary of notable features at each locus examined. **C.** Mechanisms of *URA3* inactivation. Heterozygous diploid strains carrying a single copy of *hisG-URA3-hisG* (blue circle; *URA+*) were grown under specified conditions and selected for 5FOA resistance. “Molecular events” summarize possible mechanisms of *URA3* inactivation. Co-segregation of *ura3* chromatids at mitosis would lead to a 5FOA^R^ colony (pink circle) that no longer carries the *hisG-URA3-hisG* allele. The resulting diploid would carry either the “pop-out” and a single copy of WT (i, ii), two copies of WT (iii, iv), or just one copy of WT (v, vi). L and R; the immediate upstream and downstream sequences of each insertion locus utilized for targeted introduction of the tester construct via homologous recombination (Figure S1). INT: the allele containing the tester construct.

**Figure 2:**
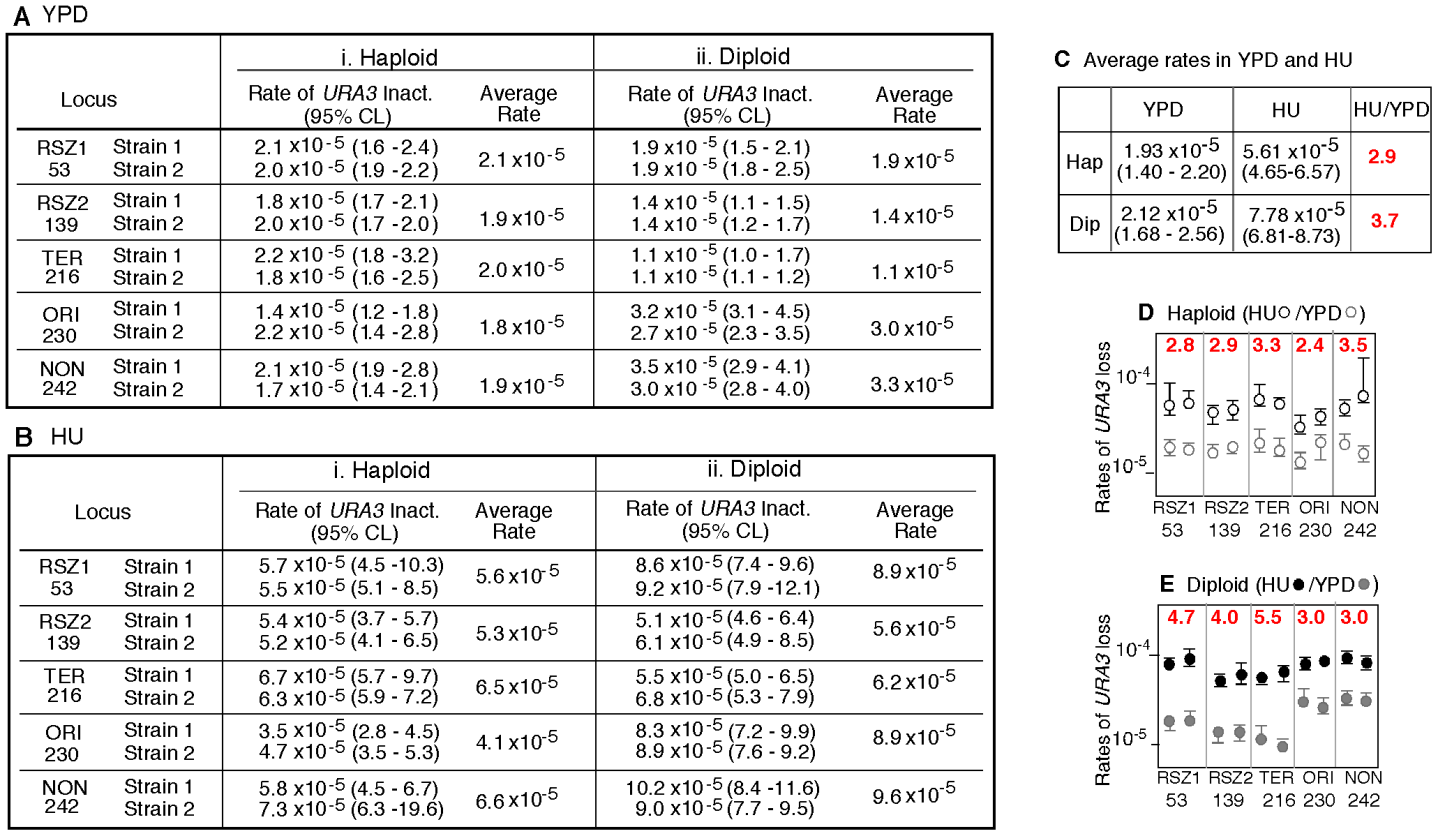
Rate of *URA3* inactivation in YPD and in HU. **A, B.** Rate of *URA3* inactivation at the indicated locus in WT haploid- or diploid- strains grown in YPD (A) or in 10 mM HU (B). 95% Confidence Limits (CLs) for each value were calculated as previously described [47]. For each locus, rate measurements from two independently derived strains were obtained by the methods of median (Figure S2) [24]. **C.** The average recombination rate (and 95% CLs) of the haploid- and diploid- strains analyzed in **A** and **B.** “HU/YPD”: The effects of HU over YPD on recombination rate was expressed as the ratio between the two average rates. **D, E.** Effects of HU on rate of *URA3* inactivation at each locus. Graphic representation of the data presented in **A** and **B.** Black and grey circles correspond to rate measurements in HU and YPD, respectively. Capped lines indicate 95% CLs. The red number at the top of each box is the ratio between the average rate values in HU and YPD at the indicated locus; the extent increase conferred by HU was statistically significant at every locus (Chi square test, p<0.05).

**Figure 3:**
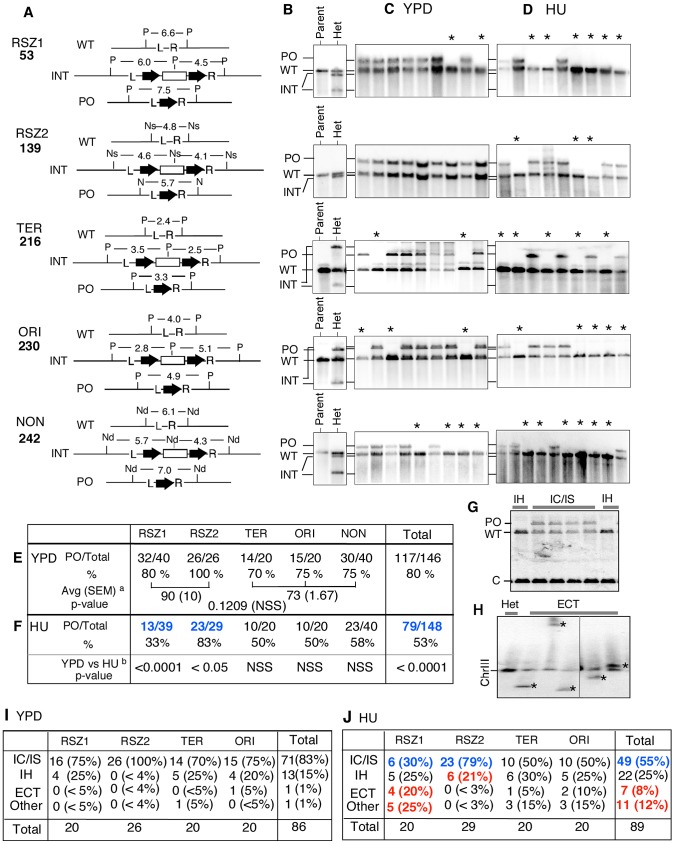
Intrachromatid and intersister (IC/IS) bias in *URA3* inactivation. **A.** For each locus examined, Southern Blot analysis was utilized to monitor the presence of three relevant alleles; (i) WT, (ii) the allele containing the *hisG-URA3-hisG* reporter integrated, hereupon referred to as “INT” for “integrant”, and (iii) the “pop-out” or “PO” allele, indicative of an intrachromatid or intersister (IC/IS) recombination or recombination related event (Figure 1C). Genomic DNA samples were digested by the indicated restriction enzymes and Southern Blot analysis was performed using the L and the R sequences as a probe (Figure S3). The numbers represent expected sizes of WT, INT, and PO fragments in kb. P: Pst1, Ns: Nsi1, Nd: Nde1. *hisG* and *URA3* sequences are represented by a block-arrow and open rectangle, respectively. **B.** Representative Southern Blot images of the “Parent” strain (*ura3*) carrying two copies of WT allele and a heterozygote or “Het”, derived from the parent carrying one WT and one INT allele at the specified locus. Indicated are the migrating positions for WT, INT, and PO fragments described in panel A. **C**, **D.** Representative images of Southern Blot analyses on 5FOA^R^ colonies that arose in YPD or HU. All 5FOA^R^ samples examined had lost the INT-fragments, suggesting that *URA3* loss in each case was likely to be mediated by one of the mechanisms described in Figure 1B rather than a base pair substitution or small deletion or insertion. * Samples that have lost the INT fragment, but also lacking the diagnostic PO fragment, suggestive of a non IC/IS mediated *URA3* inactivation event (See text). **E**, **F.** Fraction of 5FOA^R^ colonies at each locus that had undergone *URA3* inactivation via an IC/IS mediated event. For each locus, the number of 5FOA^R^ samples exhibiting the diagnostic PO fragment and the total number analyzed are shown. ^a^ The average PO fraction of the two RSZs and the three non-RSZ loci. SEM: standard error of the mean. Unpaired student's t test was performed to determine whether the difference between the two averages was statistically significant. ^b^ Fischer's exact test was utilized to determine whether the extent of reduction in PO fraction conferred by HU at each locus was statistically significant. Numbers in blue indicate statistically significant reduction. NSS: Not Statistically Significant. **G.** A representative image of Southern Blot analyses showing 5FOA^R^ colonies that had undergone *URA3* inactivation via IH recombination. The occurrence of IH mediated events (either gene conversion or crossing over; Figure 1B) was confirmed by the lack of PO band and a two fold increase in the signal associated with the WT band relative to an IC/IS sample. To assess the WT copy number, the amount of radioactive signal associated with the WT-fragment in each sample was quantified and normalized to the signal found in a control band (“C”) in the same lane (Materials and Methods). **H.** A representative image of Southern Blot image of a pulse field gel (PFG), where the occurrence of ectopic events (ECT) in each sample was inferred by the presence of a novel chromosome size fragment that hybridized with *hisG* probe, a part of the *hisG-URA3-hisG* construct (*). **I**, **J.** Frequencies of IC/IS, IH, ECT, and other mechanisms of *URA3* inactivation in 5FOA^R^ colonies. Numbers in blue and red in panel **J** denote statistically significant decrease and increase conferred by HU compared to YPD, respectively.

A *URA3* cassette flanked by short *hisG* direct repeats (*hisG-URA3-hisG*) was used [23] (Figure 1A; Figure S1). The rate of *URA3* inactivation, inferred from the acquisition of resistance to 5FOA (hereupon referred to as 5FOA^R^), was estimated based on the methods of the median [24] (Figure S2; Figure 2). The nature of genetic alteration associated with *URA3* inactivation in each 5FOA^R^ colony was determined by Southern Blot analysis (Figure S3; Figure 3A). Possible mechanisms of *URA3* inactivation in a diploid carrying a single copy of the tester construct are summarized in Figure 1C, excluding base pair alternations and small structural changes.

*URA3* inactivation via either intrachromatid or intersister (IC/IS) recombination or recombination related process such template switching would result in the allele referred to as “pop-out” or “PO”, where the *URA3* and one of the two *hisG* repeats “pop-out”, leaving only a single copy of the *hisG* (Figure 1Ci and ii). *URA3* inactivation via interhomolog (IH) gene conversion or crossing over would lead to a loss of heterozygosity (LOH), where the WT allele replaces the reporter construct: the resulting 5FOA^R^ colony would now carry two WT copies (Figure 1Ciii, iv). Ectopic recombination resulting in a translocation or chromosome loss would also lead to a LOH; however, unlike the IH-mediated LOH, a 5FOA^R^ colony resulting from the latter is expected to carry only a single copy of WT allele (Figure 1Cv, vi).

In haploid strains, *URA3* inactivation via IH recombination cannot occur, and chromosome loss would be lethal. As such, the majority of *URA3* loss events in haploids is expected to be via IC/IS mediated events.

#### Loci analysed

The reporter was integrated into two RSZ and three non RSZ loci in chromosome III (ChrIII) via homologous recombination (Materials and Methods). Notable features associated with each locus include replication origins, replication termination zones, Ty elements, tRNA sequences, and the distance from the centromere (Figure 1A and 1B). 53 kb and 139 kb correspond to the approximate midpoints of an RSZ, each comprising about 10 kb wide. All six RSZs published to date, including the 53 kb and 139 kb, are found at or near a replication termination zone (Figure 1A) [3]. In addition, the RSZ at 139 kb is near a Ty element and several tRNA sequences. The three non RSZ loci chosen were 216 kb, 230 kb, and 242 kb, corresponding to a replication termination zone, a replication origin, and a ‘no feature’ locus, respectively (Figure 1A, B). Hereupon, the five loci are referred to as RSZ1, RSZ2, TER, ORI, and NON.

#### Tester strains

Two independent haploid strains carrying a single copy of the tester construct at one of the five loci were obtained by transformation, followed by sporulation and selection (Materials and Methods). These haploid strains were used to construct corresponding diploid strains in a WT background as well as both haploid and diploid strains in an *sml1Δ, mec1Δ sml1Δ*, or *rrm3Δ*, backgrounds. Control experiments confirmed that the presence of the construct did not impact fitness of the strains examined nor the RSZ-specific chromosome fragility following thermal inactivation of Mec1 (Figure S4).

### RSZs are not recombination hotspots during unchallenged proliferation

The observations that stalled- and collapsed- replication forks can promote recombination [6], [7], [25], [26] and that RSZs are loci of slowed replication fork progression during normal S-phase [3], suggest that RSZs might be recombination hotspots during normal proliferation. (NB: In the current context, an arrested fork that can ultimately resume replication without intervention is referred to as a “stalled fork” whereas that requires an active fork-restart process is referred to as a “collapsed fork”). To test this, we compared the rate of *URA3* inactivation at the RSZ and non RSZ loci under a standard yeast growth condition (2% glucose at 30°C, hereupon referred to as “YPD”). For each locus, the rate was estimated by the method of the median from two independently derived strains, and the average of the two was used for the locus-to-locus comparison (Figure S2) [24].

In haploids, the locus specific recombination rates varied very little, from 1.8×10^−5^ per cell generation at ORI to 2.1×10^−5^ at RSZ1 (Figure 2Ai). In diploids, the variation was greater, about 3 fold, and ranged from 1.1×10^−5^ per cell generation at TER to 3.3×10^−5^ at NON (Figure 2Aii). Importantly, the rate of *URA3* inactivation at the two RSZs was not higher than the three non RSZ loci in either haploids or diploids. We conclude that RSZs are not spontaneous recombination hotspots, defined as loci of intrinsically higher recombination activities.

### RSZs are not recombination hotspots in HU

Chromosome breakage at RSZs does not occur during normal proliferation, but is promoted by a modest level of HU, high temperature, and/or inactivation of Mec1 [3], [21]. Thus, it is possible that the putative recombination hotspot activity associated with RSZs might also require these conditions. To test this, we examined the effect of HU. The same ten haploid and ten diploid strains analyzed above were subjected to a transient (18 hour) exposure to 10 mM HU before 5FOA selection. We found that the exposure lead to a statistically significant increase in rate of *URA3* inactivation at every locus (Figure 2D). Importantly, the rates at the two RSZs were not any higher than the three non RSZ loci in either haploids or diploids (Figure 2B). We conclude that RSZs are not recombination hotspots even under a condition that promotes RSZ specific chromosome breakage.

### Intrachromatid and intersister (IC/IS) bias in mitotic recombination

Next, we examined the nature of genetic alternations associated with *URA3* inactivation in diploids. To this end, we utilized Southern Blot analysis that enabled us to monitor the presence of the following three alleles (Figure 3A, Figure S3); (i) WT, (ii) the allele containing the *hisG-URA3-hisG* reporter integrated, hereupon referred to as “INT” for “integrant”, and (iii) the “pop-out” or “PO” allele, indicative of an intrachromatid or intersister (IC/IS) recombination or recombination related event (Figure 1C). As expected, the parent *ura3* diploid strain exhibited a single band diagnostic of the WT allele at each of the five loci examined; in contrast, each of the *URA3* heterozygotes derived from the parent exhibited an additional band(s), corresponding to the INT allele (Figure 3B, Figure S3).

We found that all 5FOA^R^ colonies examined had lost the INT fragment, suggesting that *URA3* inactivation in every case involved a relatively large structural change(s) at the integration locus (Figure 3C; data not shown). Overall, ∼80% (117/146) of the samples exhibited the diagnostic PO band, indicating that on average, *URA3* inactivation was four times more likely to occur via an IC/IS-mediated event than all other mechanisms combined (Figure 3E “Total”). The latter is consistent with previous reports on strong IC/IS-bias in mitotic recombination (e.g. [27]). Locus specific PO fraction ranged from 70% (14/20) at TER to 100% (26/26) at RSZ2, suggesting that the extent of IC/IS bias might be influenced by local environment (Figure 3E). The average PO fraction for the two RSZs was higher than the three non-RSZ loci (90% versus 73%; Figure 3E); however the difference was not statistically significant (p = 0.1209). We conclude that recombination activity at RSZs during standard growth condition is comparable to non RSZ loci with regard to both the rate of recombination and the extent of the IC/IS bias.

### HU down-regulates IC/IS-bias in mitotic recombination

The same Southern Blot analysis was performed on 5FOA^R^ colonies that arose in the presence of HU. Similarly to the YPD samples, all 148 HU 5FOA^R^ samples had lost the diagnostic INT fragment (Figure 3D; data not shown). Overall, 53% (79/148) of the HU 5FOA^R^ colonies carried the PO allele, a significant reduction from the 80% (117/148) observed in YPD (Figure 3F “Total”; Chi square test, p<0.0001).

The negative effect of HU on IC/IS bias was observed at every locus; however, the only statistically significant reduction was at the two RSZs (Fisher's exact test; Figure 3F). At RSZ1, the PO fraction was reduced from 80%in YPD to 33% in HU, indicating that the 4∶1 bias toward IC/IS mediated *URA3* inactivation in YPD was completely lost in HU, where the majority of *URA3* inactivation occurred via non IC/IS mediated events. RSZ2 was notable because the extent of IC/IS bias in YPD appeared to be unusually strong (26/26; Figure 3E). In HU, we found that six of 29 had undergone a non IC/IS mechanism of *URA3* loss, suggesting that the negative effect of HU might be irrespective of the intrinsic robustness of the IC/IS-bias.

### HU promotes *URA3* inactivation via interhomolog (IH), ectopic (ECT), and other mechanisms

In the current analysis, a 5FOA^R^ colony exhibiting only the WT band (e.g. Figure 3CD, samples denoted by an “*”) was inferred to have undergone *URA3* inactivation via a non IC/IS mediated event, such as an IH gene conversion/crossover, ectopic recombination (ECT), or chromosome loss (Figure 1C). As mentioned above, it is possible to distinguish an IH event from the others by the virtue of the fact that a 5FOA^R^ colony that arose via an IH recombination event would carry two copies of WT allele, while the rest carries just one (Figure 1C).

Indeed, quantitative analysis of the WT fragments in Southern Blot images revealed that some of the non IC/IS samples had a two-fold greater signal associated with the WT band relative to an IC/IS sample that carried a copy of the WT and the PO allele each (Figure 3G, compare WT band intensity in “IH” and “IC/IS” lanes); these samples were inferred to have undergone *URA3* inactivation via IH-recombination. We were also able to confirm the occurrence of an ectopic (ECT) event by the presence of a novel chromosome sized fragment containing the *hisG* sequence on a Southern Blot of a pulse field gel (PFG) (e.g. Figure 3F), suggestive of a chromosome translocation event. Applying these criteria, we were able to infer that the majority of 5FOA^R^ colonies that arose in YPD (85/86) or in HU (78/89) had undergone *URA3* inactivation via one of these three (IC/IS, IH, or ECT) mechanisms (Figure 3I and J). The rest, 1/86 in YPD and 11/89 in HU, was classified as “Other”, which would include mechanisms such as chromosome loss.

Of the 86 YPD 5FOA^R^ colonies analyzed, about 25%, or 15, had undergone *URA3* loss via a non IC/IS event(s). Among them, all but two were mediated by IH recombination. The remaining two corresponded to an ECT and an Other event each (Figure 3I, “Total”). In HU, a reduction in the fraction of IC/IS event was accompanied by a significant increase in the fractions of ECT and Other, where they rose to 8% (7/89) and 12% (11/89) from about 1% in YPD (Figure 3J, “Total”). The only significant increase in IH fraction conferred by HU was at RSZ2, where it rose from 0/26 in YPD to 6/29 in HU (Figure 3I and J).

### *sml1Δ* promotes IC/IS bias in mitotic recombination

**Figure 4:**
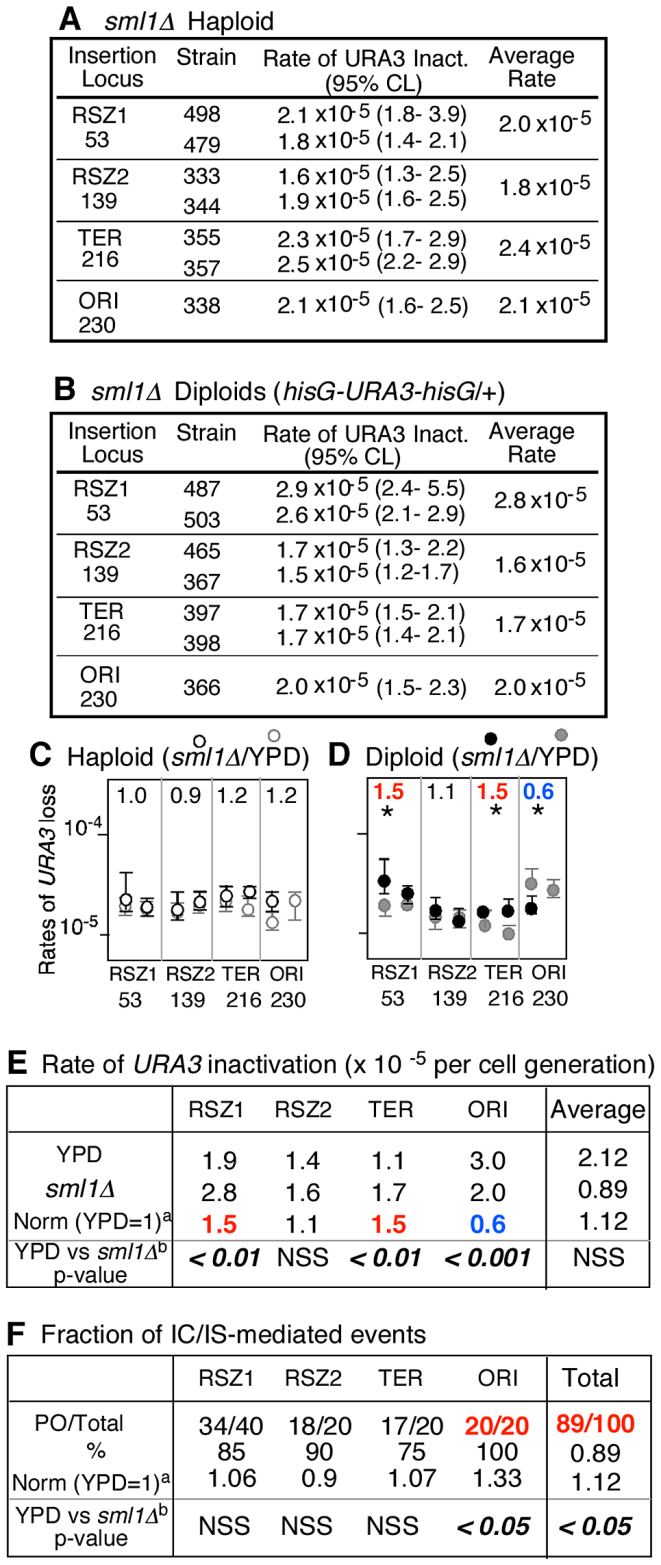
*sml1Δ* regulation of mitotic recombination. **A, B.** Rates of *URA3* inactivation at the indicated locus in *sml1Δ* haploid and diploid strains grown in YPD. 95% Confidence Limits (CLs) for each value were calculated as previously described [47]. For each locus except for ORI, rate measurements from two independently derived strains were obtained by methods of median (Figure S2) [24]. **C, D.** Effects of *sml1Δ* on rate of *URA3* inactivation. Black and grey circles correspond to rate measurements in *sml1Δ* (panels **A** and **B**) and WT (Figure 2A), respectively. Capped lines indicate 95% CLs. The number at the top of each box is the ratio between average rate values in *sml1Δ* and WT at each locus. * denotes a statistically significant change (Chi square test, p<0.05). Numbers in red and blue denote statistically significant increase or decrease, respectively. **E.** Locus specific effects of *sml1Δ* on rate of *URA3* inactivation. ^a^ The average rate at the indicated locus in *sml1Δ* was normalized to the corresponding value in WT (Figure 2). Numbers in red and blue denote statistically significant increase or decrease, respectively (Chi square test, p<0.05). NSS: Not Statistically Significant. **F.** Effect of *sml1Δ* on IC/IS bias. Fraction of 5FOA^R^ colonies that had undergone *URA3* inactivation via an IC/IS mediated event ^a^ The fraction in *sml1Δ* at each locus was normalized to the corresponding value in WT (Figure 3E). ^b^ Statistical analysis (Fischer's exact test) was performed on the effects of *sml1Δ*. The number in red denotes statistically significant increase.

To test whether the effect of HU on the IC/IS bias might have been due to reduced dNTP levels, we examined the effect of *sml1Δ*. Sml1 is a negative regulator of RNR and its deletion leads to a ∼2.5 fold increase in dNTP levels [22]. We found that the mutation conferred a modest, but statistically significant increase in the overall PO fraction from 80% (117/146) in YPD to 89% (89/100) in *sml1Δ* (Chi-square analysis, p<0.05; Figure 4F, “Total”). The observed effects of HU and *sml1Δ* suggest a positive correlation between dNTP levels and the extent of IC/IS bias, and implicate dNTP availability in regulation of recombination outcomes and genome rearrangements.

The results also revealed that the effect of *sml1Δ* on IC/IS bias might be locus dependent; while the overall effect was an increase, the mutation actually lead to a modest decrease in the bias at RSZ2 from 100% (26/26) in WT to 90% (18/20) in *sml1Δ* (Figure 3E; Figure 4F). Similarly, we found that the effect of *sml1Δ* on recombination rate was locus specific; while the mutation lead to an increase at RSZ1, RSZ2, and TER, but it reduced the rate at ORI (Figure 4D, E). Overall, the effect of *sml1Δ* on ORI differed from the rest in that it was the only locus where the mutation conferred a statistically significant reduction in recombination rate and a significant increase in the IC/IS-bias. It would be require analysis of additional origin sequences to confirm whether the observed effect might be origin specific.

No significant effect of *sml1Δ* was observed on the rate of *URA3* inactivation in haploids (Figure 4A, C).

### Negative correlation between recombination activity and IC/IS bias in *URA3* inactivation

If chromosome breakage is mechanistically linked to recombination activity, then HU, a condition that promotes RSZ specific chromosome breakage, should also confer an RSZ specific increase in the rate of *URA3* inactivation. Unexpectedly however, we found that HU increased the rate at both RSZ and non RSZ loci, indicating that the breakage was not linked to recombination activity. To confirm this further, we examined the effects of additional conditions shown to regulate chromosome breakage at RSZs. Specifically, we chose high temperature and *mec1Δ sml1Δ*, the two conditions shown to promote the breakage, and *rrm3Δ*, a mutation shown to suppress it [21]. Rrm3 encodes a DNA helicase involved in replication fork progression through ∼1000 discrete fork pause sites in the budding yeast genome, whose inactivation leads to fork stalling at these loci [28], [29]. The *rrm3Δ* mediated fork stalling triggers Mec1/Tel1 dependent S phase checkpoint activation and Sml1 removal. The latter in turn, promotes fork progression through RSZ and prevents RSZ breakage even in the absence of Mec1 function [21], [30]. We limited our analyses to diploids only, where the effect on IC/IS bias can be assessed in addition to the rate of *URA3* inactivation.

**Figure 5:**
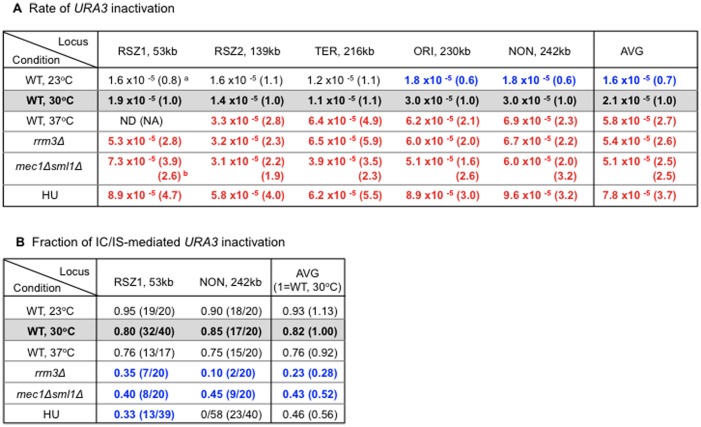
Regulation of endogenous mitotic recombination. **A.** Regulation of recombination rate. Rate of *URA3* inactivation at each locus in different genetic backgrounds and growth conditions. The numbers represent average rate value at each locus based on two independent rate measurements (Materials and Methods). ^a^ Each value is normalized to the corresponding WT value from YPD 30°C shown in blue box. Numbers in red and blue denote statistically significant increase or decrease, respectively. ^b^ For the *mec1Δ sml1Δ* measurements, normalization was also performed using the corresponding values in a *sml1Δ* background. **B.** Regulation of IC/IS-bias. Fraction of 5FOA^R^ colonies exhibiting the PO fragment indicative *URA3* inactivation via IC/IS-recombination at RSZ1 and NON. Blue numbers denote statistically significant decrease compared to the WT 30°C values. ^a^ Fisher's exact test was performed to assess whether the differences between the PO fractions at RSZ1 and NON observed in *rrm3Δ* and HU were statistically significant. P values for the *rrm3Δ* and HU values were 0.064 and 0.018, respectively, indicating that only the differences in HU was significant.

Results showed that high temperature (37°C), *rrm3Δ*, and *mec1Δ sml1Δ* increased the average rate of *URA3* inactivation by ∼2.5 fold over WT at 30°C (Figure 5A, “Average”). Low temperature (23°C), on the other hand, lead to ∼30% reduction. These observations confirmed previous reports on recombinogenic effects of *rrm3Δ* and *mec1Δ sml1Δ*. Furthermore, they revealed a positive correlation between temperature and recombination rate, suggesting that temperature itself might regulate endogenous recombination activity. Importantly, the observed effect was not specific to RSZs, providing further support for the notion that chromosome fragility and recombination activity at RSZs are not mechanistically linked.

Similarly to HU, the three recombinogenic conditions, 37°C, *rrm3Δ*, and *mec1Δ sml1Δ* lead to a reduction in the overall PO fraction (Figure 5B, “Average”). In contrast, low temperature (23°C), the only condition that decreased the rate of recombination, lead to a modest increase in the fraction. Together, these observations suggest an association between increased recombination activity and loss of IC/IS bias. Finally, results also showed that the effects of different conditions on IC/IS bias can be either RSZ specific, as in the case of HU, or general, as in the case of temperature, *rrm3Δ*, and *mec1Δ sml1Δ* (Figure 5B legend).

## Discussion

RSZ is a homolog of the mammalian common fragile sites, noted for its sensitivity to dNTP depletion and inactivation of Mec1 and Tel1, the budding yeast ATR and ATM, respectively. Here, we utilized RSZs to address the proposal that mammalian fragile sites are recombination hotspots and that increased recombination activity at these loci contributes to cancer development. Our analyses revealed that; (i) RSZs are not recombination hotspots, (ii) recombinogenic conditions (e.g. *rrm3Δ*, *mec1Δ*, HU, and high temperature) down-regulate IC/IS-bias in mitotic recombination, and (iii) RSZs exhibit greater sensitivity to HU dependent loss of IC/IS bias. Below, we discuss each of these findings further.

### RSZs are not recombination hotspots

We found that rates of *URA3* inactivation at RSZs were comparable to non-RSZ loci under all tested conditions. Based on this, we conclude that RSZs are not recombination hotspots, defined as loci of increased recombination activity. A key assumption is that the *URA3* inactivation event monitored in the current study is a readout for recombination activities at the locus, and not for an indirect effect(s) of a more distant element(s). For example, studies of LOH in diploids indicate that most LOH events occur by a crossover between the heterozygous loci, and that the frequency of these events increases as a function of distance from centromere (e.g. [31]). In addition, Ty elements are recombination hotspots that might affect recombination activities downstream of the loci (e.g. [32]). Notably however, the frequency of the LOH events monitored in the current study was independent of the distance from CENIII (Figure 3I, J). Similarly, the rates of overall *URA3* inactivation at the loci downstream (the three non RSZ loci) and upstream (the two RSZ loci) of a Ty element were comparable (Figure 2B, 4B, and 5A). Furthermore, the approach employed in the current study – i.e. integration of a reporter construct at a specific locus - is a widely utilized means of assessing local recombination activities (e.g. [33]). Together, these considerations strongly suggest that the assay utilized in the current study monitors local recombination activity.

It was surprising that RSZs did not have intrinsically higher rates of recombination than non RSZs. However, the current observation is actually consistent with the fact that RSZ breakage occurs during prometaphase in the context of topoisomerase II- and condensin- dependent chromosome condensation [3], [20], while most spontaneous mitotic recombination occurs during S phase in the context of stalled- and collapsed-replication forks [5], [7], [26]. The apparent temporal separation and differential genetic requirements suggest that chromosome breakage and recombination at RSZs might each entail a process that is independently regulated. However, it is also possible that the lack of correlation is a feature specific to RSZ (and mammalian fragile sites, by extension) and not a general feature of a fragile site.

### Regulation of endogenous mitotic recombination rate

Our results confirmed an earlier observation that the locus-to-locus variation in spontaneous mitotic recombination rate is relatively modest, in contrast to the variation in meiotic recombination rates, which can be several orders of magnitude [34], [35]. This difference is likely due, at least in part, to the nature of DNA structure that leads to recombination in each case: stalled- or collapsed-replication forks for mitotic recombination and developmentally programmed DSBs for meiotic recombination [26], [36]. Formation of meiotic DSBs are regulated at multiple levels, including targeted localization of Spo11, the enzyme that catalyzes the breakage, to the hotspot regions of the genome [37]. This in turn ensures that meiotic DSBs do not occur uniformly throughout the genome, but preferentially at DSB hotspots. In contrast, the occurrence of stalled- or collapsed-forks leading to spontaneous mitotic recombination does not appear to be regulated per se; rather, it appears to be a unintended consequence of a replication fork encountering a locus that is difficult to replicate, for example, due to either unusual DNA or chromatin structures or damaged DNA [6], [7], [25], [26], [38].

Previous studies have shown that genes encoding for proteins involved in processes such as replication fork progression, stalled fork integrity, and fork restart impact endogenous mitotic recombination rates (e.g. [39], [40]). The recombinogenic effects of HU or *rrm3Δ* observed in current study are likely due to increased incidences (either in the frequency and/or the duration) of fork stalling stemming from depletion of dNTP pools or loss of a replisome associated helicase activity, respectively [28], [41]. The effects of *mec1Δ sml1Δ*, on the other hand, is unlikely due to increased fork stalling because replication forks proceed faster in an *sml1Δ* background compared to WT [3]. Given that Mec1 is required for stability of stalled forks and is a key regulator of homologous recombination and recombination related processes involved in replication fork restart [18], [42], the recomginogenic effects of *mec1Δ sml1Δ* are likely to stem from a defect(s) in processes that occur after fork stalling. The mechanism(s) by which temperature might affect endogenous recombination activity remains unknown. Notably however, there has been a precedent in meiotic recombination, where temperature appears to play an important regulatory role(s) [43].

### Regulation of IC/IS bias in mitotic recombination

On average, *URA3* inactivation under the standard growth condition was four times more likely to occur via an IC/IS mediated event than all other mechanisms combined. We found that HU, high temperature, *rrm3Δ*, and *mec1Δ sml1Δ* abolished this bias while low temperature and *sml1Δ* enhanced it. These observations suggest that partner choice in mitotic recombination might be subjected to regulation, for example by factors like dNTP availability and temperature. During meiotic recombiantion, a significant fraction of meiotic DSBs is developmentally programmed to be repaired with an IH bias, using an intact homolog as a repair template, rather than a sister chromatid. Evidence indicates that such IH bias in meiotic recombination is mediated, at least in part, by expression of several meiosis specific chromosomal proteins that fundamentally alter meiotic chromatin structure; this in turn, favours physical interaction between the homologs while minimizing that between sister chromatids, overcoming the IC/IS bias intrinsic during mitotic recombination [44], [45], [46]. The latter implicates chromatin structure, notably the status of sister chromatid cohesion, in mitotic IC/IS bias.

### Fragile sites and cancer

We utilized RSZ, a model for mammalian common fragile sites, to address the proposal that the fragile sites contribute to cancer due to increased recombination activity at the loci. The evidence presented here, however, suggests an alternative mechanism, which implicates the nature of recombination outcomes, rather than the overall recombination rate. The only RSZ specific recombination activity revealed in the current study was its greater sensitivity to HU induced loss of IC/IS bias. Although the sample size is limiting (i.e. one insertion in each of two RSZs), an implication would be that, depending on the nature of stress, recombination events at RSZs (and at mammalian common fragile sites, by extension), might be more likely to lead to a LOH and translocation, the type of alterations shown to contribute to cancer. Taken together, current observations provide a fresh insight into the ways in which fragile sites and other recombinogenic conditions may contribute to genome rearrangements.

## Materials and Methods

### Yeast strains

Relevant genotypes of the strains utilized in current study are summarized in Table S1. All *URA3* strains were generated by standard yeast genetics procedures including transformation, mating, sporulation, and specific phenotype selections. A wild type haploid strain NKY291, was transformed with each of the five DNA fragments containing the *hisG-URA3-hisG* cassette flanked by ∼500 bp upstream (L) and ∼500 bp downstream (R) genomic sequences of the targeted loci. The fragments were prepared from integration plasmids (Figure S1) by NotI digestion and gel purification. Correct integration of *hisG-URA3-hisG* at each locus among randomly selected *URA* transformants was confirmed by Southern Blot analysis (Figure S3). Two independent transformants of each locus were mated with NKY292, a WT haploid strain of the opposite mating type to generate heterozygous diploid strains, from which *URA3* haploids were re-derived. These haploid strains (JDCY 463, 465, 230, 233, 239, 243, 232, 233, 235, 237) were used for all subsequent strain construction.

### Rate of *URA3* inactivation

The rate of *URA3* inactivation was determined by the method of median [24]. For each measurement, 15 colonies of comparable size (1.5–2 mm in diameter) freshly grown on YPD plates, were individually suspended in 5 mls of YPD or YPD+10 mM HU and cultured for 18 hours at 30°C (Figure S2A). Samples were then diluted in water and plated on YPD or 5FOA plates to measure the number of total viable cells or those that had undergone a *URA3* inactivation event, respectively. To ensure that only the *URA3* inactivation events that occurred during the 18 hour of unselected growth in liquid medium were included for the analyses, only the 5FOA^R^colonies of comparable size (1.5–2 mm) were counted following a three day incubation at 30°C. Statistical analyses on the rates of *URA3* inactivation were assessed as described [47].

### Molecular characterization of *URA3*-loss in 5FOA^R^ recombinants

For each condition examined, a total of 20 or more independent 5FOA^R^ colonies from each locus were subjected to molecular analysis (Figure S2B). Genomic DNA from each colony was restricted using the appropriate restriction enzyme (Figure S3; Figure 3) and subjected to Southern Blot analysis. For each locus, the L and R fragments used for plasmid construction (Figure S1) were used as probes. As a control for quantifying the relative signals associated with different diagnostic fragments (e.g. Figure 3E), a PCR amplified Mec1 fragment corresponding to nucleotide numbers 5539 to 7027 in the OFR was included as a probe (Table S2). Each restriction enzyme used for Southern analysis cleaves endogenous *MEC1* sequence to generate a novel sized fragment that hybridizes to the *MEC1* probe. To confirm the occurrence of ectopic recombination events, the candidate 5FOA^R^ colonies were analyzed by Pulse Field Gel electrophoresis (PFGE) and Southern Blot analyses using *hisG* as a probe. For PFGE, Chromosome sized genomic DNA samples were prepared in low melting point agarous plugs as previously described [48]. Electrophoresis condition optimized for resolution around ChrIII was performed as described [49].

## Supporting Information

Figure S1 Construction of *hisG-URA3-hisG* integration cassettes. The aim was to create five plasmids, each containing the tester cassettes flanked by the sequences immediately upstream (“L”) and downstream (“R”) of one of the five loci examined. These plasmids were referred to as pJDCX-hUh (panel E), where X denotes the insertion locus, 53, 139, 216, 230, or 242 kb (Table S2). A. The L and the R fragments of each locus were PCR amplified from genomic DNA of NKY291 (Table S1) using two sets of primers, F1/R1 and F2/R2, respectively (Table S2). The 5′-ends of F1 and R2 contain a Not1 restriction site, that were used to excise the fragment used for transformation. F2 and R1 tail sequences contain a BglII and are complementary to each other. B. L and R fragments from each locus were purified and used as a PCR template. The reaction was carried out using primers F1 and R2. The complementary sequences introduced to the tails of F2 and R2 (above) enabled generation of a contiguous fragment that contained a BglII site at the junction between L and R. C. The fusion PCR product was cloned into the NotI site of Bluescript. D. The BamHI/BglII fragment containing *hisG-URA3-hisG* from pNKY51 (Table S2) was cloned into the BglII site to generate the pJDCX-hUh plasmids (E), from where the NotI fragments used for transformation were purified.
(PDF)

Figure S2 Analysis of rate and nature of genetic alterations of *URA3* inactivation. Each strain was patched onto YPG from ^−^80°C glycerol stock. Following 17 hour incubation at 30°C, cells were streaked for single colonies on YPD. After 55 hour incubation, 15 colonies of comparable size (1.5–2 mm in diameter) were used to inoculate 15 flasks containing 5 ml YPD with or without 10 mM HU. Appropriate dilutions of these cultures were plated onto YPD or 5FOA plates following 18 hour incubation. The numbers of colonies on YPD or 5FOA plates were counted two or three days after, respectively. ^a^ For Southern Blot analysis, one colony from ten or more 5FOA plates were examined per strain, or 20 or more in total per condition. The only exception was for the ORI analysis in an *sml1Δ* background (Figure 4F), where 20 independent colonies from 20 parallel YPD liquid cultures from the same strain were analyzed.
(PDF)

Figure S3 Southern Blot analysis of *URA3* haploid and diploid strains in a WT background utilized in the current study. Depicted in each panel are schematics of DNA structures resolved using the probes indicated by dashed lines. “WT” and “INT” represent the locus analyzed before and after insertion of *hisG-URA3-hisG*, respectively. “L” and “R” are the ∼500 bp genomic DNA sequences used for generating each targeting construct (Figure S1). Probes were PCR amplified using the primers listed in Table S2. Vertical arrows mark relevant restriction enzyme (RE) sites; (A), StuI; (B), StuI and XhoI; (C), ApaI; (D), ApaI and PmeI; and (E), StuI. * in panel D is a non-specific cross hybridizing band.
(PDF)

Figure S4 Effects of *hisG-URA3-hisG* insertion on fitness and RSZ-fragility. A, B. Fitness of haploid- and diploid- strains in a WT or *sml1Δ* background carrying a single copy of *hisG-URA3-hisG* at on the five loci examined. The box on the upper right indicates the strains spotted onto the plates. For each locus, fitness of two independently derived strains was assessed. As a control, WT and *mec1Δ sml1Δ* strains without the reporter were used. Ten-fold serial dilutions of exponentially growing cells were spotted onto YPD or YPD containing 10 mM HU. The plates were photographed after 3 day incubation at 30°C. C. Pulse Field Gel Electrophoresis (PFGE) followed by Southern Blot analysis using *YCR098* as a probe detects linear chromosome fragments of various lengths extending from the labelled end to the site of chromosome breakage. RSZ positions along Chromsome III (ChrIII) were deduced from the lengths of fragmented species. This approach was used to identify six RSZs in ChrIII referred to as I through VI [5]. D. Haploid strains carrying a single copy of *hisG-URA3-hisG* at each of the five loci were used to construct a set of *mec1-40^ts^* strains with *hisG-URA3-hisG* (Table S1). The resulting *mec1-40^ts^* strains were cultured at 30°C overnight and then at 37°C for five hours. Chromosome samples were prepared and subjected to PFGE/Southern Blot analysis as described in panel C [5]. The roman numerals on the left correspond to the RSZ numbers in panel C.
(PDF)

Table S1 Yeast Strains utilized in current study. All *URA3* strains are derived from NKY291 and NKY292 using standard yeast genetics protocols. NKYs are from Nancy Kleckner's laboratory (Harvard University). JDCYs are those created during this study and are also *ho::LYS2, ura3, lys2, leu2::hisG*.
(PDF)

Table S2 Yeast Strains utilized in current study. A. The primers beginning with a specific number are those utilized for constructing targeting constructs and also as probes for Southern Blot analysis for each locus described in Figure 3B–D. The Mec1 primers are used for preparing a probe for an internal control fragment in Figure 3G. B. pBS and pNKY51 were used to construct the pJDCX-hUh plasmids containing fragments used for transformation. See Figure S1 legend for further information.
(PDF)
