# Supplementary figures and images for: Recombinogenic Conditions Influence Partner Choice in Spontaneous Mitotic Recombination
Source: PLoS Genet. 2013 Nov 7;9(11):e1003931. doi: 10.1371/journal.pgen.1003931 (PMC3820797; doi:10.1371/journal.pgen.1003931)

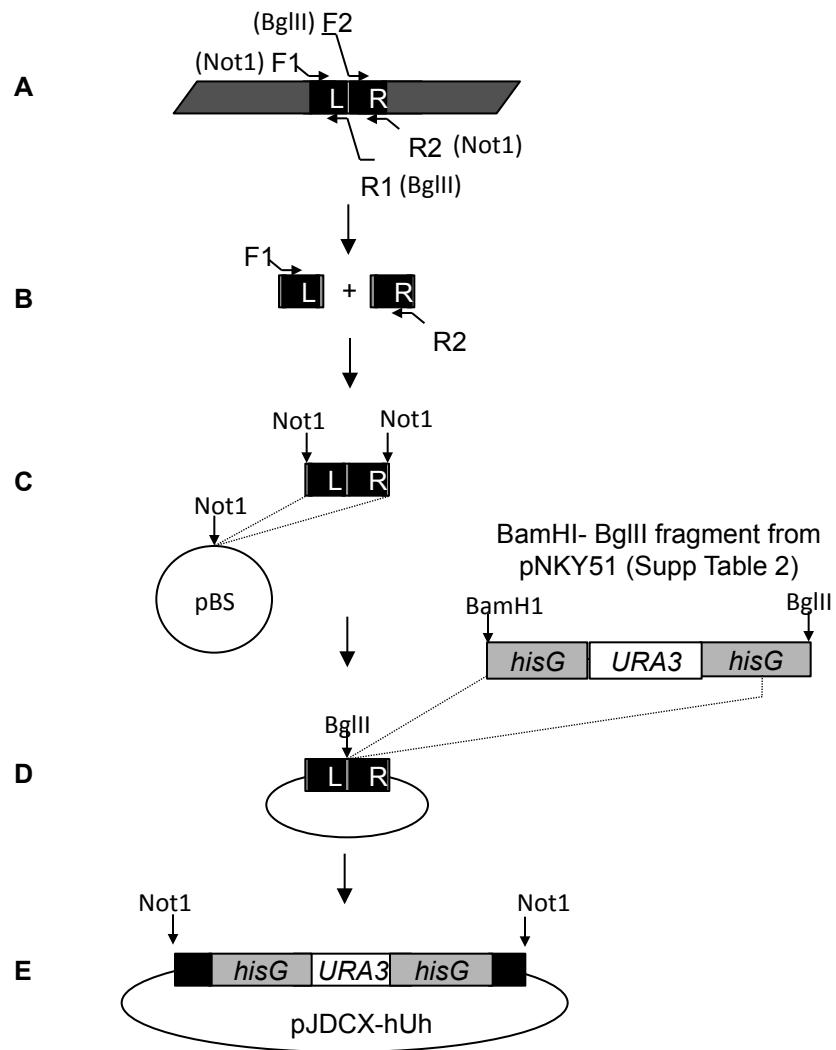

Supplement: Figure S1 — Construction of hisG-URA3-hisG integration cassettes. The aim was to create five plasmids, each containing the tester cassettes flanked by the sequences immediately upstream (“L”) and downstream (“R”) of one of the five loci examined. These plasmids were referred to as pJDCX-hUh (panel E), where X denotes the insertion locus, 53, 139, 216, 230, or 242 kb (Table S2). A. The L and the R fragments of each locus were PCR amplified from genomic DNA of NKY291 (Table S1) using two sets of primers, F1/R1 and F2/R2, respectively (Table S2). The 5′-ends of F1 and R2 contain a Not1 restriction site, that were used to excise the fragment used for transformation. F2 and R1 tail sequences contain a BglII and are complementary to each other. B. L and R fragments from each locus were purified and used as a PCR template. The reaction was carried out using primers F1 and R2. The complementary sequences introduced to the tails of F2 and R2 (above) enabled generation of a contiguous fragment that contained a BglII site at the junction between L and R. C. The fusion PCR product was cloned into the NotI site of Bluescript. D. The BamHI/BglII fragment containing hisG-URA3-hisG from pNKY51 (Table S2) was cloned into the BglII site to generate the pJDCX-hUh plasmids (E), from where the NotI fragments used for transformation were purified. (PDF) [file pgen.1003931.s001.pdf]

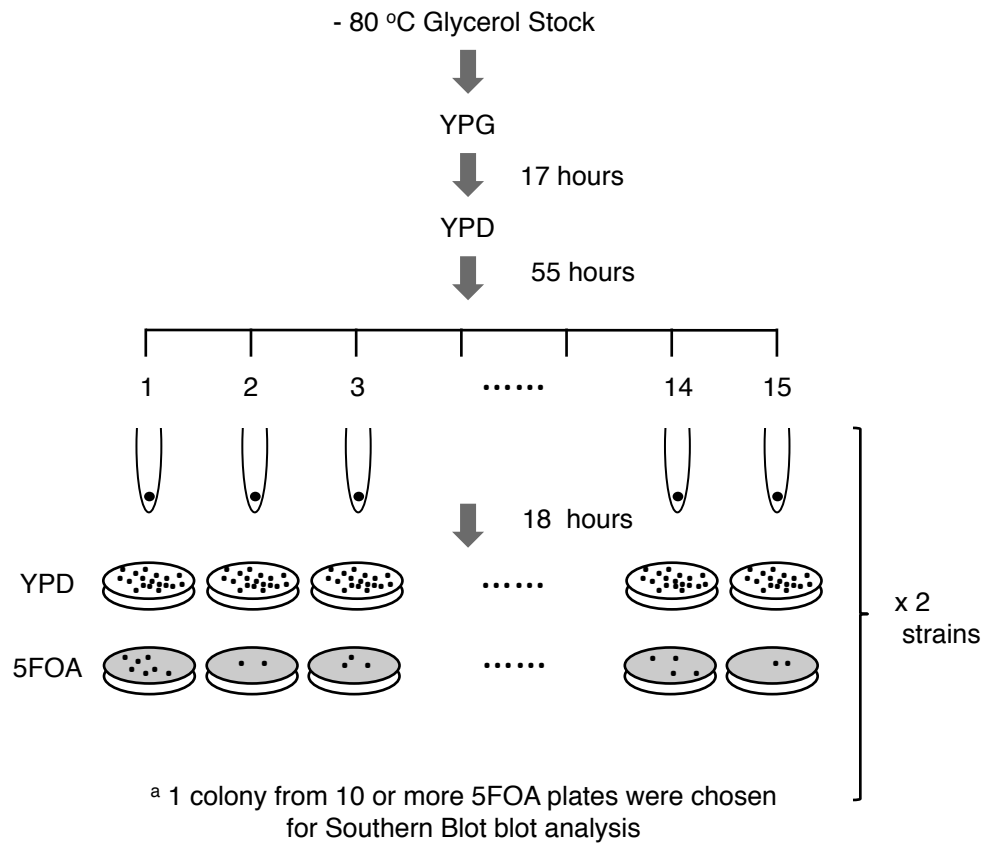

Supplement: Figure S2 — Analysis of rate and nature of genetic alterations of URA3 inactivation. Each strain was patched onto YPG from −80°C glycerol stock. Following 17 hour incubation at 30°C, cells were streaked for single colonies on YPD. After 55 hour incubation, 15 colonies of comparable size (1.5–2 mm in diameter) were used to inoculate 15 flasks containing 5 ml YPD with or without 10 mM HU. Appropriate dilutions of these cultures were plated onto YPD or 5FOA plates following 18 hour incubation. The numbers of colonies on YPD or 5FOA plates were counted two or three days after, respectively. a For Southern Blot analysis, one colony from ten or more 5FOA plates were examined per strain, or 20 or more in total per condition. The only exception was for the ORI analysis in an sml1Δ background (Figure 4F), where 20 independent colonies from 20 parallel YPD liquid cultures from the same strain were analyzed. (PDF) [file pgen.1003931.s002.pdf]

**A. 53kb**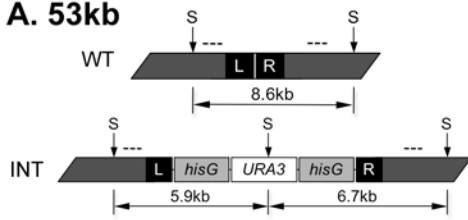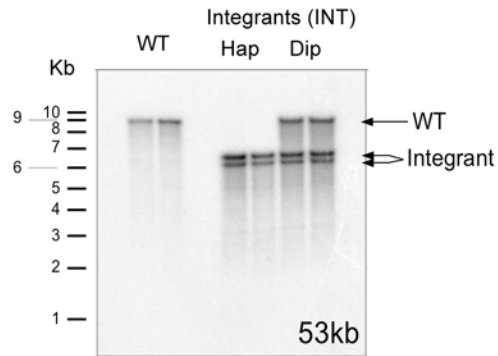**B. 139kb**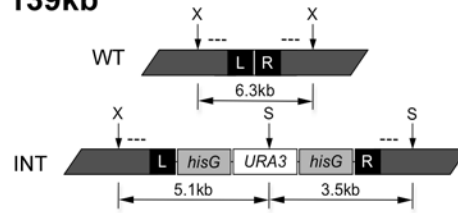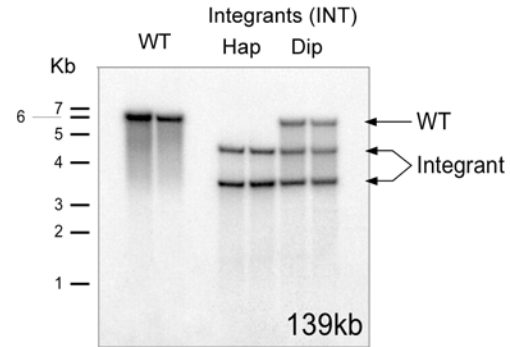**C. 216kb**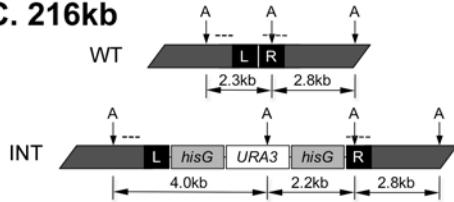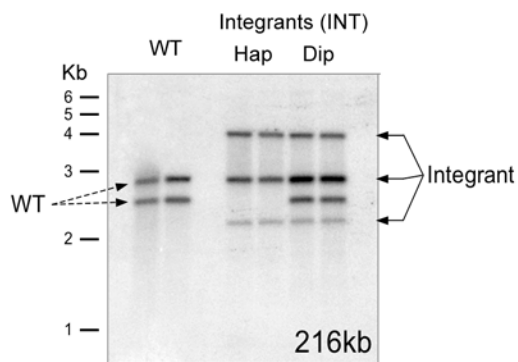**D. 230kb**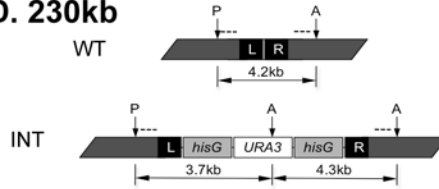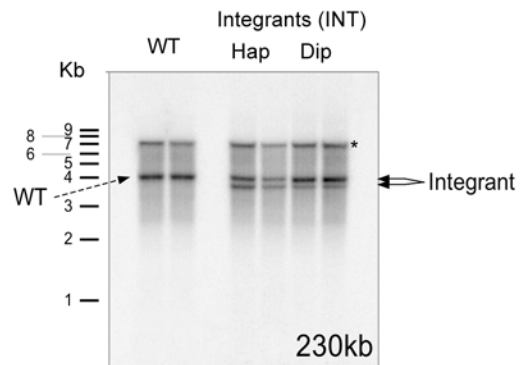**E. 242kb**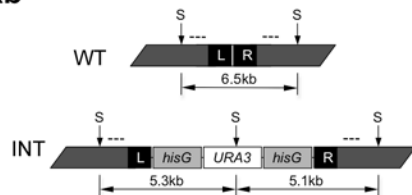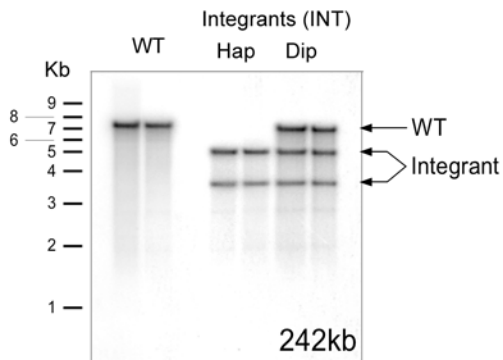

Supplement: Figure S3 — Southern Blot analysis of URA3 haploid and diploid strains in a WT background utilized in the current study. Depicted in each panel are schematics of DNA structures resolved using the probes indicated by dashed lines. “WT” and “INT” represent the locus analyzed before and after insertion of hisG-URA3-hisG, respectively. “L” and “R” are the ∼500 bp genomic DNA sequences used for generating each targeting construct (Figure S1). Probes were PCR amplified using the primers listed in Table S2. Vertical arrows mark relevant restriction enzyme (RE) sites; (A), StuI; (B), StuI and XhoI; (C), ApaI; (D), ApaI and PmeI; and (E), StuI. * in panel D is a non-specific cross hybridizing band. (PDF) [file pgen.1003931.s003.pdf]

## A Haploids

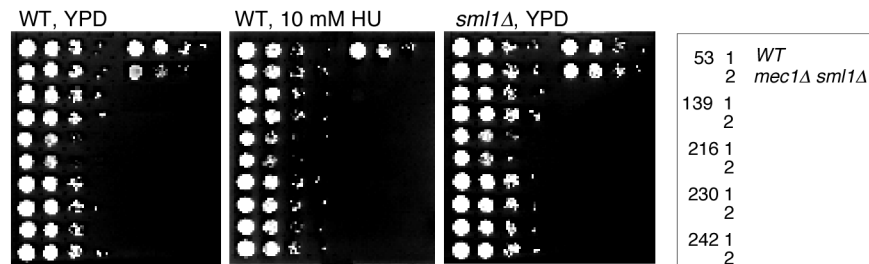

## B Diploids

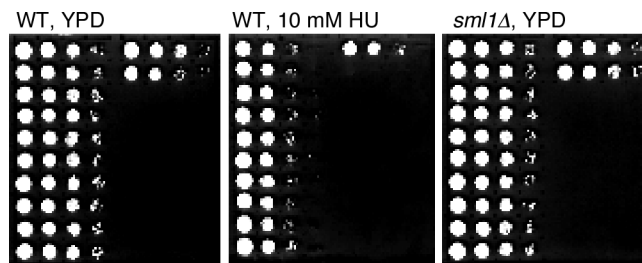

Key

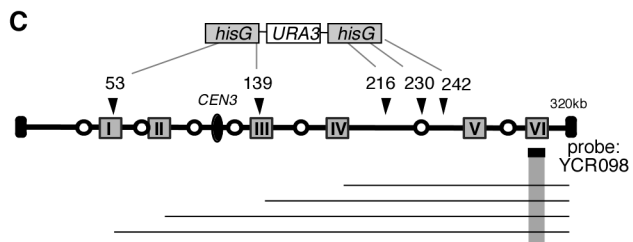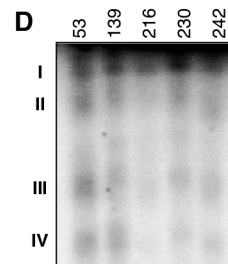

Supplement: Figure S4 — Effects of hisG-URA3-hisG insertion on fitness and RSZ-fragility. A, B. Fitness of haploid- and diploid- strains in a WT or sml1Δ background carrying a single copy of hisG-URA3-hisG at on the five loci examined. The box on the upper right indicates the strains spotted onto the plates. For each locus, fitness of two independently derived strains was assessed. As a control, WT and mec1Δ sml1Δ strains without the reporter were used. Ten-fold serial dilutions of exponentially growing cells were spotted onto YPD or YPD containing 10 mM HU. The plates were photographed after 3 day incubation at 30°C. C. Pulse Field Gel Electrophoresis (PFGE) followed by Southern Blot analysis using YCR098 as a probe detects linear chromosome fragments of various lengths extending from the labelled end to the site of chromosome breakage. RSZ positions along Chromsome III (ChrIII) were deduced from the lengths of fragmented species. This approach was used to identify six RSZs in ChrIII referred to as I through VI [5]. D. Haploid strains carrying a single copy of hisG-URA3-hisG at each of the five loci were used to construct a set of mec1-40ts strains with hisG-URA3-hisG (Table S1). The resulting mec1-40ts strains were cultured at 30°C overnight and then at 37°C for five hours. Chromosome samples were prepared and subjected to PFGE/Southern Blot analysis as described in panel C [5]. The roman numerals on the left correspond to the RSZ numbers in panel C. (PDF) [file pgen.1003931.s004.pdf]
