# Supplementary material for: Recombinogenic Conditions Influence Partner Choice in Spontaneous Mitotic Recombination
Source: PLoS Genet. 2013 Nov 7;9(11):e1003931. doi: 10.1371/journal.pgen.1003931 (PMC3820797; doi:10.1371/journal.pgen.1003931)
Supplement: Table S1 — Yeast Strains utilized in current study. All URA3 strains are derived from NKY291 and NKY292 using standard yeast genetics protocols. NKYs are from Nancy Kleckner's laboratory (Harvard University). JDCYs are those created during this study and are also ho::LYS2, ura3, lys2, leu2::hisG. (PDF) [file pgen.1003931.s005.pdf]

Supplementary Table 1. Yeast strains utilized in current study

Strain	MAT	Relevant genotype
NKY291	**a**	*ho:: LYS2, ura3, lys2, leu2::hisG*
NKY292	α	*ho:: LYS2, ura3, lys2, leu2::hisG*
JDCY463, 465	**a**	*ChrIII-53k::hisG-URA3-hisG*
JDCY230, 231	**a**	*ChrIII-139k::hisG-URA3-hisG*
JDCY239, 243	**a**	*ChrIII-216k::hisG-URA3-hisG*
JDCY232, 233	**a**	*ChrIII-230k::hisG-URA3-hisG*
JDCY235, 237	**a**	*ChrIII-242k::hisG-URA3-hisG*
JDCY479, 498	**a**	*sml1Δ::HphMx4, ChrIII-53k::hisG-URA3-hisG*
JDCY333, 344	**a**	*sml1Δ::HphMx4, ChrIII-139k::hisG-URA3-hisG*
JDCY355, 357	**a**	*sml1Δ::HphMx4, ChrIII-216k::hisG-URA3-hisG*
JDCY329, 338	**a**	*sml1Δ::HphMx4, ChrIII-230k::hisG-URA3-hisG*
JDCY350, 293	**a**	*sml1Δ::HphMx4, ChrIII-242k::hisG-URA3-hisG*
JDCY469, 477	**a/α**	*ChrIII-53k::hisG-URA3-hisG/+*
JDCY179, 181	**a/α**	*ChrIII-139k::hisG-URA3-hisG/+*
JDCY199, 203	**a/α**	*ChrIII-216k::hisG-URA3-hisG/+*
JDCY185, 524	**a/α**	*ChrIII-230k::hisG-URA3-hisG/+*
JDCY189, 526	**a/α**	*ChrIII-242k::hisG-URA3-hisG/+*
JDCY487, 503	**a/α**	*sml1Δ::HphMx4/”, ChrIII-53k::hisG-URA3-hisG/+*
JDCY365, 367	**a/α**	*sml1Δ::HphMx4/”, ChrIII-139k::hisG-URA3-hisG/+*
JDCY397, 398	**a/α**	*sml1Δ::HphMx4/”, ChrIII-216k::hisG-URA3-hisG/+*
JDCY366, 376	**a/α**	*sml1Δ::HphMx4/”, ChrIII-230k::hisG-URA3-hisG/+*
JDCY491, 492	**a/α**	*rrmΔ::HphMX4/”, ChrIII-53k::hisG-URA3-hisG/+*
JDCY407, 429	**a/α**	*rrmΔ::HphMX4/”, ChrIII-139k::hisG-URA3-hisG/+*
JDCY406, 524	**a/α**	*rrmΔ::HphMX4/”, ChrIII-216k::hisG-URA3-hisG/+*
JDCY400, 411	**a/α**	*rrmΔ::HphMX4/”, ChrIII-230k::hisG-URA3-hisG/+*
JDCY191	**a/α**	*rrmΔ::HphMX4/”, ChrIII-242k::hisG-URA3-hisG/+*
JDCY486, 499	**a/α**	*mec1Δ:: LEU2/, sml1Δ::HphMx4/”, ChrIII-53k::hisG-URA3-hisG/+*
JDCY374, 375	**a/α**	*mec1Δ:: LEU2/, sml1Δ::HphMx4/”, ChrIII-139k::hisG-URA3-hisG/+*
JDCY395, 396	**a/α**	*mec1Δ:: LEU2/, sml1Δ::HphMx4/”, ChrIII-216k::hisG-URA3-hisG/+*
JDCY427	**a/α**	*mec1Δ:: LEU2/, sml1Δ::HphMx4/”, ChrIII-230k::hisG-URA3-hisG/+*
JDCY311, 394	**a/α**	*mec1Δ:: LEU2/, sml1Δ::HphMx4/”, ChrIII-242k::hisG-URA3-hisG/+*
