# Supplementary material for: Recombinogenic Conditions Influence Partner Choice in Spontaneous Mitotic Recombination
Source: PLoS Genet. 2013 Nov 7;9(11):e1003931. doi: 10.1371/journal.pgen.1003931 (PMC3820797; doi:10.1371/journal.pgen.1003931)
Supplement: Table S2 — Yeast Strains utilized in current study. A. The primers beginning with a specific number are those utilized for constructing targeting constructs and also as probes for Southern Blot analysis for each locus described in Figure 3B–D. The Mec1 primers are used for preparing a probe for an internal control fragment in Figure 3G. B. pBS and pNKY51 were used to construct the pJDCX-hUh plasmids containing fragments used for transformation. See Figure S1 legend for further information. (PDF) [file pgen.1003931.s006.pdf]

Supplementary Table 2. Primers and plasmids utilized in current study

A. Primers

Primer	Primer sequence (5'-3')
53k_F1	GGG GCG GCC GCA AGG TGC ACT TGA AGA TTG C
53k_R1	AGA GTG CAT GCA GAT CTA TGT ATC AGT AAT TTA GTG AGC
53k_F2	TAC ATA GAT CTG CAT GCA CTC TAA AGG AGC TGG GC
53k_R2	GGG GCG GCC GCG TCG ACA GTT CTC TCA AGG
139k_F1	GGG GCG GCC GCT CAG TAT ATA ATG ATC TTT TGC
139k_R1	TTA TAG CAT GCA GAT CTG TTT TGT AAG AAA TTA TAC TTC C
139k_F2	AAA ACA GAT CTG CAT GCT ATA ACA AGA TCG TCT AAG TAA C
139k_R2	GGG GCG GCC GCC GTA CGT TAG TGA AAC ATC C
216k_F1	GGG GCG GCC GCG ATG GTT TTT CTC TTG GTG G
216k_R1	AGA GTG CAT GCA GAT CTT TCA TTC TAA GAA AGA AGG AGC
216k_F2	ATG AAA GAT CTG CAT GCA CTC TTG GCA GAC TCC TTG
216k_R2	GGG GCG GCC GCT CTT AGT AGA GAG CTG GAG G
230k_F1	GGG GCG GCC GCA ATA CTG CAA ATA CCA AAT ACC
230k_R1	TAG CGG CAT GCA GAT CTA ATT ATA CAA TAG ACA AGA CC
230k_F2	TAA TTA GAT CTG CAT GCC GCT AGT ATT TGT TTT GCA TTG
230k_R2	GGG GCG GCC GCT TAC GGA CGA AAT ACA AAT GC
242k_F1	GGG GCG GCC GCA AGG TGC ACT TGA AGA TTG C
242k_R1	AGA GTG CAT GCA GAT CTA TGT ATC AGT AAT TTA GTG AGC
242k_F2	TAC ATA GAT CTG CAT GCA CTC TAA AGG AGC TGG GC
242k_R2	GGG GCG GCC GCG TCG ACA GTT CTC TCA AGG
Mec1_F_21	GGC CAG CAA CAC TCA ATA CCA G
Mec1_R_23	AAT TTG CCC AAA CTA ATC ACG

B. Plasmids

Plasmid	Relevant details
pBS	Bluescript
pNKY51	*hisG-URA3-hisG* cassette
pJDC53-hUh	*ChrIII 52758::hisG-URA3-hisG* in pBS
pJDC139-hUh	*ChrIII 144606::hisG-URA3-hisG* in pBS
pJDC216-hUh	*ChrIII 210805:hisG-URA3-hisG* in pBS
pJDC230-hUh	*ChrIII 225492::hisG-URA3-hisG* in pBS
pJDC242-hUh	*ChrIII 236450::hisG-URA3-hisG* in pBS
